# Development, acceptability, appropriateness and appeal of a cancer clinical trials implementation intervention for rural- and minority-serving urology practices

**DOI:** 10.1186/s13063-019-3658-z

**Published:** 2019-10-07

**Authors:** Shellie Ellis, Mugur Geana, Tomas Griebling, Charles McWilliams, Jessie Gills, Kelly Stratton, Christine Mackay, Ariel Shifter, Andrew Zganjar, Brantley Thrasher

**Affiliations:** 1 Department of Population Health, University of Kansas School of Medicine, 3901 Rainbow Blvd., MS 3044, Kansas City, KS 66160 USA; 2 School of Journalism and Mass Communications, University of Kansas, Lawrence, KS USA; 3 Department of Urology and The Landon Center on Aging, University of Kansas School of Medicine, Kansas City, KS USA; 4 Department of Urology, University of Oklahoma Health Sciences Center, Oklahoma City, OK USA; 5 Department of Urology, Louisiana State University Health Sciences Center, New Orleans, LA USA; 6 Department of Urology, University of Kansas School of Medicine, Kansas City, KS USA

**Keywords:** Implementation science, Design for dissemination, Health care delivery, Clinical practice guidelines, Specialty care, Cancer clinical trials, Acceptability, Innovation adoption, Urology, Rural health disparities

## Abstract

**Background:**

Few community urologists offer cancer patients the opportunity to participate in cancer clinical trials, despite national guidelines that recommend it, depriving an estimated 260,000 urological cancer patients of guideline-concordant care each year. Existing strategies to increase urologists’ offer of clinical trials are designed for resource-rich environments and are not feasible for many community urologists. We sought to design an implementation intervention for dissemination in under-resourced community urology practices and to compare its acceptability, appropriateness and adoption appeal among trial-naïve and trial-experienced urologists.

**Methods:**

We used a design-for-dissemination approach, informed by the Theoretical Domains Framework and Behavior Change Wheel, to match determinants of the clinical trial offer to theoretically informed implementation strategies. We described the implementation intervention in evaluation workshops offered at urology professional society meetings. We surveyed participants to assess the implementation intervention’s acceptability and appropriateness using validated instruments. We also measured adoption appeal, intention to adopt and previous trial offer.

**Results:**

Our design process resulted in a multi-modal implementation intervention, comprised of multiple implementation strategies designed to address six domains from the Theoretical Domains Framework. Evaluation workshops delivered at four meetings, convened five separate professional societies. Sixty-one percent of those offered an opportunity to participate in the implementation intervention indicated intention to adopt. Average implementation intervention acceptability and appropriateness ratings were 4.4 and 4.4 (out of 5), respectively. Acceptability scores were statistically significantly higher among those offering trials compared to those not (*p* = 0.03). Appropriateness scores did not differ between those offering trials and those not (*p* = 0.24). After urologists ranked their top three innovation attributes, 43% of urologists included practice reputation in their top three reasons for offering clinical trials; 30% listed practice differentiation among their top three reasons. No statistically significant differences were found between those who offered trials and those who did not among any of the innovation attributes.

**Conclusions:**

LEARN|INFORM|RECRUIT is a promising implementation intervention to address low accrual to clinical trials, poised for implementation and effectiveness testing. The implementation intervention is appealing to its target audience and may have equal uptake among trial-naïve and trial-experienced practices.

**Electronic supplementary material:**

The online version of this article (10.1186/s13063-019-3658-z) contains supplementary material, which is available to authorized users.

## Background

Participation rates in clinical trials to develop new cancer therapies have been directly linked to improvements in population-level outcomes for the subgroups of patients who participate in them [1]. However, only about 8% of cancer patients participate in trials, thwarting the development of new therapies [2]. A study conducted through the National Cancer Institute’s (NCI’s) National Clinical Trials Network found that 18% of cancer trials closed with low accrual or achieved less than 50% of their target enrollment at 3 years or more after the start of the trial [5]. In contrast, a large majority of cancer patients report willingness to participate [6–8], but may never be offered the opportunity. Many cancer clinical trials are conducted at National Cancer Institute-designated cancer centers, academic centers and select community oncology practices supported by the NCI [9]. Yet relatively few cancer patients are treated at these sites where clinical trials are available, resulting in less than half of all cancer patients having access to trials [2, 10, 11]. These structural limitations in how cancer care is delivered have recently been identified as the most important factor leading to low patient participation [2].

Structural constraints in cancer care delivery are particularly limiting for *urological* cancer patients’ access to clinical trials. Urological cancer accounts for one in every five newly diagnosed cancers in the US each year, involving approximately 330,000 patients [12]. Most of these cancers are diagnosed, and treatment paths established, by urologists, without input from medical oncologists to whom clinical trial infrastructure is most often directed [13]. Although multidisciplinary care is common in the treatment of some cancers [14, 15], multidisciplinary care in urological cancers is less well established, and particularly rare in community settings, where 80% of urological cancer patients are treated [1, 14, 16–18]. Consequently, relative to other cancer patients, approximately 260,000 urological cancer patients may be less likely to be offered clinical trials at the point of treatment decision-making and their treatment plans are less likely to include the systematic clinical trial consideration that is often part of multidisciplinary case review [19–21]. Despite interventions which reach into the community to increase access to cancer clinical trials [16, 22–26], community urologists report little awareness of the urological cancer trials available, even those available in their local community. They are less likely to report access to clinical trials than their academic counterparts [13], and few report offering clinical trials to patients [26]. To address the structural challenges which limit cancer patients’ access to clinical trials, thereby slowing the development of effective cancer therapies, the American Cancer Society in 2018 convened a national committee to make recommendations for overcoming these hurdles [2, 28]. That committee ranked development of new strategies targeting non-research sites to refer interested patients to trial opportunities as a top priority [28].

Unfortunately, little research exists to guide the development of implementation strategies aimed at research-naïve providers. To date most research on physician referral to clinical trials has focused on physicians at institutions already engaged in research [6, 29–33]. However, physicians with access to clinical trials may have needs that are different than those who do not [34–36]. For example, community urologists experienced in clinical trial referral describe perceived barriers among their less experienced peers that they know do not, in fact, exist in practice, suggesting that different strategies may be required at different points across the adoption to implementation continuum. For example, strategies needed to facilitate adoption may need to address perceptions of the potential impact on practice (e.g., the myth of “losing patients”), whereas those needed to facilitate implementation may need to address memory and attention through reminders and workflow integration. Thus, it is important to evaluate potential implementation strategies, distinguishing these two levels of experience.

Research-naïve urologists in community practice have reported willingness to try strategies to facilitate the offer of cancer clinical trials in their practice, but also report potential barriers in their ability to do so. Previous research based on the Theoretical Domains Framework (TDF) [37] has suggested that the offer of clinical trials may be influenced by constructs in select TDF domains: environmental resources; social influences; knowledge; memory, attention and decision processes; social/professional role and identity; and beliefs about consequences. Thus, an implementation intervention addressing these behavioral determinants may be effective in increasing the reach of clinical trials. Because the determinants were identified in rural-serving practices in a single state, the degree to which the implementation intervention would appeal to a broader group of urologists, particularly those in other rural-serving communities and minority-serving communities, was unknown. The present study describes the development and appeal of an implementation intervention addressing these behavioral determinants.

### Objective

The objective of this study was to apply an implementation science approach to develop an implementation intervention to increase urology practices’ referral to cancer clinical trials and to compare the acceptability, appropriateness and appeal of the approach between urologists naïve to clinical trials and those experienced in offering clinical trials across a diverse group of urology practices in the South and Midwest United States.

## Methods

We used a design-for-dissemination approach [38–40] informed by the Behavior Change Wheel [41] to develop an implementation intervention to address behavioral determinants of offering clinical trials. We then presented the implementation intervention to a diverse sample of urologists and surveyed them to evaluate the implementation intervention’s acceptability, appropriateness and appeal. The Institutional Review Board of a Midwestern university reviewed and approved the study.

### Implementation intervention development

**Fig. 1:**
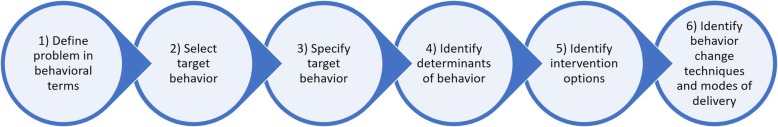
Six steps of implementation intervention development

We assembled a transdisciplinary team of urologists, implementation scientists, education technologists and communications experts to increase the likelihood that the resulting approach would be adopted and used in practice [38–40]. We followed the six steps of the Behavioural Change Wheel (Fig. 1) to: (1) define the problem in behavioral terms; (2) select the target behavior; (3) specify the target behavior; (4) identify determinants of the target behavior; (5) identify intervention options; and (6) match behavioral-change techniques and mode of intervention delivery to the intended users’ context [42]. The Behaviour Change Wheel is derived from a distillation of 19 frameworks of behavior change through systematic literature review [42]. As others have noted, the process was iterative [43], resulting in our final intervention package and implementation strategies.

For Steps 1–3, we conducted narrative review of the available literature, relied on interviews with urologists about their decision-making processes in cancer care [44–48], and elicited clinical experience of study team members. Semi-structured qualitative interviews of 22 academic and community US urologists were conducted at the 2015 American Urological Association (AUA) annual meeting and by telephone, subsequent to the meeting. As part of the larger study on barriers to a particular treatment, interviews were transcribed and coded and the cancer care delivery process was mapped to understand the urology practice context.

Behavioral determinants to community urologists’ offer of clinical trials, needed for Step 4, were collected according to the TDF and reported elsewhere [26]. Briefly, we conducted semi-structured interviews of urologists and urology staff in rural-serving community urology practices in Kansas. Participation was limited to practices which had urological oncology trials open locally at the time of the interview. We analyzed the data, applying template analysis using a codebook of TDF constructs. The results were also used to understand the clinical trial referral process in behavioral terms (Step 1) and context surrounding the behavior (Step 6).

For each determinant identified in the previous study as salient, we selected intervention functions or policy categories from the Behavior Change Wheel’s list of nine functions and seven policy categories (Step 5). Finally, in Step 6 we reviewed behavioral-change techniques to identify those most relevant to each function or policy and simultaneously considered the best mode of delivery to arrive at theoretically supported implementation strategies to deliver the components. Selection was informed by feasibility and opportunities in the local communities in which urologists practiced. We interviewed cancer centers in the communities of urology practices participating in the determinants study about their willingness and experience engaging urology practices. We used a strategic planning approach and assessed their responses to identify strengths, weakness, threats and opportunities for supporting urology practice efforts. Thus, feasibility of potential approaches was used to inform the identification of optimal intervention options to address practice-identified determinants of referral and to select the most potentially effective behavioral-change techniques and modes of intervention delivery.

### Implementation intervention evaluation

#### Evaluation approach

We created a workshop to present and test the appeal of the implementation intervention. The didactic workshop included evidence-based educational content about the value of clinical trials from a health care providers’ perspective, including the impact on the practice, the clinical workflow and the patient-provider relationship. Other content described the implementation intervention components. Educational content was delivered by community oncologists who were members of the local professional societies to which they were presenting and identified as influencers by organizers of the local meetings. Information about the implementation intervention was delivered by the implementation intervention developers. We organized the educational sessions in conjunction with local urology professional society meetings. Targeted urology professional societies served both state and regional professional societies. We chose multiple loco-regional venues over a single national venue as the local meetings are preferred by community urologists from smaller practices. The workshops were offered to all prospective meeting attendees at each of the meetings via letter to all urologists in the states served by state societies, and via announcement through the program booklet and meeting email communications sent to regional society members. Prospective attendees at the state meetings were offered US$50 gift cards for participation in a 1-h meeting. Prospective attendees at the regional meeting were offered CME credit for participation in a 1.5-h workshop. The evaluation workshop was delivered at four meetings, convening five separate professional societies. The societies represented urologists across 10 states in the Midwest and South. Workshop location and placement within the official meeting agenda varied.

#### Measures

Intervention appeal was evaluated via survey distributed to a purposive sample of all workshop attendees (Additional file 1). The survey instrument included three scales to assess the appeal of the implementation intervention and a single item assessing whether participants currently offer clinical trials. We used the four-item, validated scales, the *Acceptability of Intervention Measure* (AIM) [49] and the *Intervention Appropriateness Measure* (IAM) [49] to measure acceptability and appropriateness. We used a novel measure, the *Attributes of Innovation Adoption* [47] scale to assess the implementation intervention’s appeal. All items were rated on a Likert response scale ranging from 1 to 5 with higher scores indicating greater acceptability, appropriateness, or attribute appeal. Participants were asked to rank the three innovation attributes that were most important to them. We measured behavioral intent to adopt the implementation intervention by asking participants to provide contact information for follow-up, collected separately from the survey responses to protect confidentiality. Due to capacity limitations, behavioral intent was only measured when and where there was capacity to deliver the implementation intervention.

#### Analysis

To calculate the acceptability and appropriateness scores, we summed the Likert ratings and averaged them across the four items. Each innovation attribute was scored individually. Ratings greater than 3.0 were considered salient to adoption. Student’s *t* test was used to assess differences by current offer of clinical trials. We combined the top ranked innovation attributes and calculated the frequency each was included in the top three among all participants.

## Results

### The implementation intervention

#### Step 1. Define problem in behavioral terms

**Fig. 2:**
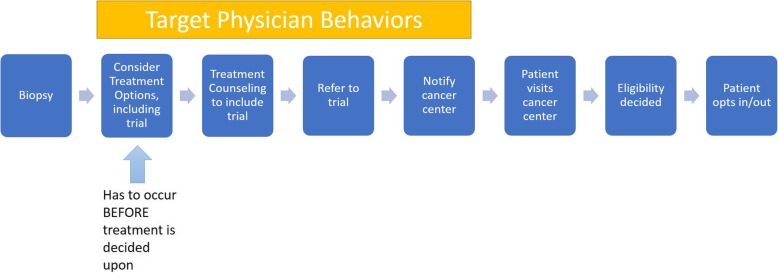
Process map to identify and select target behaviors (Step 1)

Narrative literature review suggested that patients do not participate in clinical trials due to a number of reasons. Often, they are asked too late in the process, after treatments have already been decided upon [50] or initiated [51]. Thus, ensuring that clinical trials be considered as a treatment option at the point of initial treatment decision-making is critical. Therefore, we articulated the clinical trial issue from a community urologists’ workflow (Fig. 2) to identify the ideal time to place clinical trials and provide scaffolding to understand lack of accrual from a behavioral perspective. In typical practice, potential cancer patients present for biopsy and return for treatment counseling, either in conjunction with result presentation or following telephoned results. Urologists devise a preliminary treatment recommendation upon reviewing the results, the timing of which varies by practice. This treatment recommendation is presented and discussed with the patient at a treatment counseling visit and a treatment is scheduled, sometimes with additional visits for discussion of treatment options.

The target for behavior change was identified as the urologist. Urologists and staff agree that identifying and discussing treatment options falls within the professional responsibilities of the urologist [19]. However, physicians require motivation to include trials in their assessment and awareness of what trials are available [26, 52, 53]. To incorporate trials as a treatment option into treatment counseling, they need knowledge and skills to initiate the conversation, often lacking among most physicians who have little exposure to trials in their medical training.

A structural barrier to clinical trial accrual arises because most trial participation models require a practice to undergo an intensive process to obtain human subject credentialing, and make substantial additional investments in learning about trials, opening them in their practice, and conducting extensive data collection [54]. This may be an unrealistic expectation for many providers, but particularly for community-practicing urologists focused solely on clinical service provision. Based on the willingness to engage in the low-intensity efforts we observed in our qualitative study, we reconceptualized the target behavior as referral for eligibility screening, rather than eligibility screening itself. To execute the referral, urologists need trusted referral partners to whom they can refer. They also need a mechanism by which they can efficiently communicate with the referral partner to ensure that patient care—for which they are ethically and legally responsible—is delegated appropriately [26].

#### Step 2. Select target behavior

Having defined the problem in behavioral terms, we thus selected the target behaviors as: (1) considering clinical trials as a treatment option; (2) treatment counseling inclusive of clinical trials; (3) the act of referral; and (4) cancer program communication (Fig. 2).

#### Step 3. Specify target behavior

We specified each target behavior based on our interviews with community urologists and their staff, with particular attention paid to aligning the behaviors with the professional roles and identities perceived among both physicians and staff [26]. Table 1 illustrates for each target behavior who, what, when, where, how often, and with whom each behavior should occur. For simplicity, we recommend that urologists should consider, counsel and refer *all* newly diagnosed cancer patients for clinical trial eligibility screening. Consideration should occur at review of biopsy results using the broad eligibility criteria provided by the trial expert in the normal location of review. The referral should occur at the treatment counseling visit prior to patient decision on treatment. Notification of the referral to the cancer program should occur immediately following referral and could be delegated to clinic staff.

**Table 1:** Specification of the behavior

Step 2	Step 3	
Select target behavior	Specify target behavior	
Consider	*Who?*	Urologist
	*What?*	Clinical trial as a treatment option
	*When?*	Prior to treatment decision
	*Where?*	At location of results review
	*How often?*	At each cancer diagnosis
	*With whom?*	Each diagnosed cancer patient
Counsel	*Who?*	Urologist
	*What?*	Clinical trials are a treatment option
	*When?*	At first treatment counseling visit
	*Where?*	In the exam room
	*How often?*	Every initial cancer treatment counseling visit
	*With whom?*	All patients meeting broad eligibility criteria (determined by flow sheet)
Refer	*Who?*	Urologist
	*What?*	Signal to patient and cancer program that patient is recommended for clinical trial eligibility screening
	*When?*	During and immediately following treatment counseling visit
	*Where?*	Exam room and location where charting is performed
	*How often?*	Each time clinical trial is included in treatment counseling
	*With whom?*	All patients meeting broad eligibility criteria (determined by flow sheet) who do not refuse referral
Notify	*Who?*	Urologist or designated clinic staff
	*What?*	Cancer patient is scheduling visit for clinical trial eligibility screening
	*When?*	At patient’s convenience in next 3 weeks
	*Where?*	Immediately following treatment counseling visit
	*How often?*	Each time referral is documented
	*With whom?*	All patients meeting broad eligibility criteria (determined by flow sheet) who do not refuse referral

Step 1 is illustrated in Fig. 2. Steps 4–6 are illustrated in Table 2

#### Step 4. Identify determinants of behavior

Previous work identified six of the 14 TDF domains as most salient for community urologists’ initial adoption of the four key clinical trial behaviors [19]. In particular, community urologists could be highly motivated by urology peers and professional societies to offer clinical trials. They believed that their trial involvement could accrue positive social consequences to their practice by differentiating their practice from others. They saw referral to a trial specialist as a natural alignment with the current practice of referring cancer patients to specialists in other treatment modalities (e.g., radiation oncologist). They acknowledged the need for concise trial information to consider trials as a treatment option, and brief skills training to counsel patients. They needed reminders of trial opportunities at the point of care and support for their patients, not only to prepare them for treatment counseling, but also to help them understand the referral process.

#### Step 5. Identify intervention options

We mapped each determinant to an empirically or theoretically supported intervention function or policy category from the Behavior Change Wheel. Table 2 lists the key determinants and each determinants’ COM-B category in columns 1 and 2, and the corresponding intervention functions and policies in columns 3 and 4.

**Table 2:** Mapping of behavioral determinants to intervention options and content and implementation options

Step 4		Step 5		Step 6		
Determinants of referral		Intervention options		Content and implementation options		
*COM-B*	*TDF*	*Intervention functions*	*Policy categories*	*Behavior-change techniques*	*Mode of delivery*	*Also addressed in …*
Capability	*Knowledge*	Education, training, modeling	Communication/marketing	Commitment, social support (practical), instruction on how to perform the behavior, information about social and environmental consequences, demonstration of the behavior, information about others’ approval, credible source, material reward, framing/reframing	Continuing education workshop: *Expanding Treatment Options for Urological Cancer Patients*, Professional society offering delivered by physician champion and endorsed by South Central Section of the AUA, Clinical trial flow sheet	Toolkit website
				Review outcome goals, Feedback on outcomes of behavior, social support (emotional), instruction on how to perform the behavior, information about social and environmental consequences, information about others’ approval, credible source, social reward, identification of self as role model	Quarterly Newsletter featuring AUA president endorsement, trial briefs, *Why I Do This* segment, *Meet the Cancer Center* segment, and referral and accrual audit and feedback	Continuing education workshop, newsletter, toolkit website
				Problem-solving, goal-setting, discrepancy between current behavior and goal behavior, self-monitoring of behavior, social support-unspecified, instruction on how to perform the behavior	*Talking About Trials* in-service curriculum	
				Prompts/cues, adding objects to the environment; instruction on how to perform behavior -non-specified; non-specified reward	Point-of-care interactive patient education table tent	
	*Memory, attention and decision processes*	Enablement	Service provision, environmental/social planning	Prompts/cues	Available clinical trials’ point-of-care flow sheet	
Opportunity	*Environmental resources*		Service provision	Prompts/cues, restructuring social environment	Clinical trial referral prescription pad	
				Prompts/cues, restructuring social environment	Cancer center “hotline” referral process	
				Restructuring social environment, adding objects to the environment, instruction on how to perform behavior (patient)	*What this Trial Entails* patient video series	
				Restructuring social environment, adding objects to the environment, instruction on how to perform behavior (patient)	*Participating in a Cancer Clinical Trial* patient brochure	
	*Social influences*	Persuasion, environmental restructuring	Environmental/social planning, service provision	Social support (practical), restructuring the social environment, instruction on how to perform the behavior	Meet the investigator Breakfast/lunch	Continuing education workshop, newsletter
Motivation	*Social professional role and identity*	Enablement	Environmental/social planning, guidelines	Restructuring social environment	Referral role delineation	Continuing education workshop, toolkit website
				Feedback on outcome of behavior	Cancer center eligibility screening feedback	
				Feedback on outcome of behavior, information on how to perform the behavior, information about consequences	GU Trial Chart Note	
				Generalization of target behavior	Align role delineation with perceived roles and identity	
	*Beliefs about consequences*	Incentivization, enablement	Education	Instruction on how to perform the behavior, information about social and environmental consequences, material incentive	Co-management discussion	
				Prompts/cues, social reward, non-specific incentive	Branding through stationary, pens, brochures and media	

Step 1 is provided in Fig. 2. Steps 2 and 3 are provided in Table 1

All behavior-change techniques are directed at the urology provider, unless otherwise indicated in parentheses

*AUA* American Urological Association, *COM-B* Capability, Opportunity, Motivation and Behavior Model *GU* genitourinary, *TDF* Theoretical Domains Framework

#### Step 6. Identify behavior-change techniques and implementation options

For each intervention function or policy needed, our team brainstormed behavior-change techniques and potentially effective modes of delivery. These were iteratively shaped while reviewing opportunities available in the community or within national support structures and considering the contextual determinants in community practice. Columns 5 and 6 of Table 2 list the behavior-change techniques and specific mode of delivery.

Our design process resulted in a multi-modal implementation intervention comprised of multiple implementation strategies. Together, this set of strategies is called LEARN|INFORM|RECRUIT and includes continuing education workshops, newsletters, clinical trial reminders, point-of-care materials, referral tools, referral network building and patient-support materials. It is available to practices interested in adopting the program in controlled evaluation studies at learn-inform-recruit.org. Upon enrollment, LEARN|INFORM|RECRUIT is made available as an implementation intervention tailored to each practice through personalization of trial opportunities available in the local community, development of a dedicated referral pathway to the cancer center, and practice branding of patient-facing materials. It is externally facilitated by trained study staff through a mixture of in-person, telephone and electronic communications delivered in the practice setting and supported by a non-public website of video content. Other materials are printed for the practices’ convenience and mailed to the practice at program launch and when updates are necessary (e.g., opening of new clinical trials).

### Implementation intervention evaluation

#### Response

Characteristics of the evaluation workshops, audience size and participation are presented in Table 3. Across the four workshops 67 participants registered attendance (15% of all professional society meeting attendees). Fifty-four participants across the four meetings evaluating the implementation intervention provided evaluations with no missing data (81% response rate). Among them, 78% reported currently offering trials at their practice.

**Table 3:** Evaluation workshop meeting characteristics

	Site A	Site B	Site C	Site D
Organizational sponsor scope	State	State	State	Regional
Meeting scope	1 state	2 states	1 state	8 US states, Mexico + Central America
Placement on meeting agenda	Breakout	Plenary	No	Breakout
Workshop location	On-site	On-site	Off-site	On-site
Recruitment strategy	Letter	Letter	Letter	Program + meeting PR
Number urologists invited	104	0	84	2189
Incentive	US$50	US$50	US$50	CME Credit
Total meeting attendance^a^	54	41	N/A^d^	341
Workshop attendance	10 (19%)	13 (32%)	9 (10%)	35 (10%)
Percent attendees non-academic^b^	35%	32%	67%	5%
Behavioral intent to adopt	10	–^c^	9	14

*CME* continuing medical education, *N/A* not applicable, *PR* Public relations

^a^Meeting attendance includes urologists and non-physician attendees

^b^Proportion calculated on rostered attendees where data available

^c^Not offered opportunity to adopt due to capacity limitations

^d^Data not available from Louisiana Urological Society; proportion attendance conservatively based on number of urologists invited

#### Implementation intervention appeal

Among those attending the three workshops offering an opportunity to participate in the implementation intervention, 61% (33/54) asked to be contacted to participate in the program. Potential adopters represented urology practices from eight US states and two states in Mexico. Urologists from eight of 10 US states that were targeted as part of the evaluation workshop expressed interest in adopting the program. A practice from a state not targeted also expressed interest.

#### Intervention acceptability and appropriateness

Average implementation intervention acceptability and appropriateness ratings were high: 4.4 and 4.4 (out of 5), respectively (Table 4). Acceptability scores were statistically significantly higher among those offering trials compared to those not (*p* = 0.03). Appropriateness scores did not differ between those offering trials and those not (*p* = 0.24).

**Table 4:** Ratings of acceptability and appropriateness by prior offer of clinical trials

	Average rating	Prior offer of clinical trials	No prior offer of clinical trials	Significance (*p* level)
Acceptability	4.4	4.6	4.2	*p = 0.03*
Appropriateness	4.4	4.5	4.2	*p = 0.24*
*n* = 54				

Higher scores indicate greater perceived acceptability and appropriateness

**Fig. 3:**
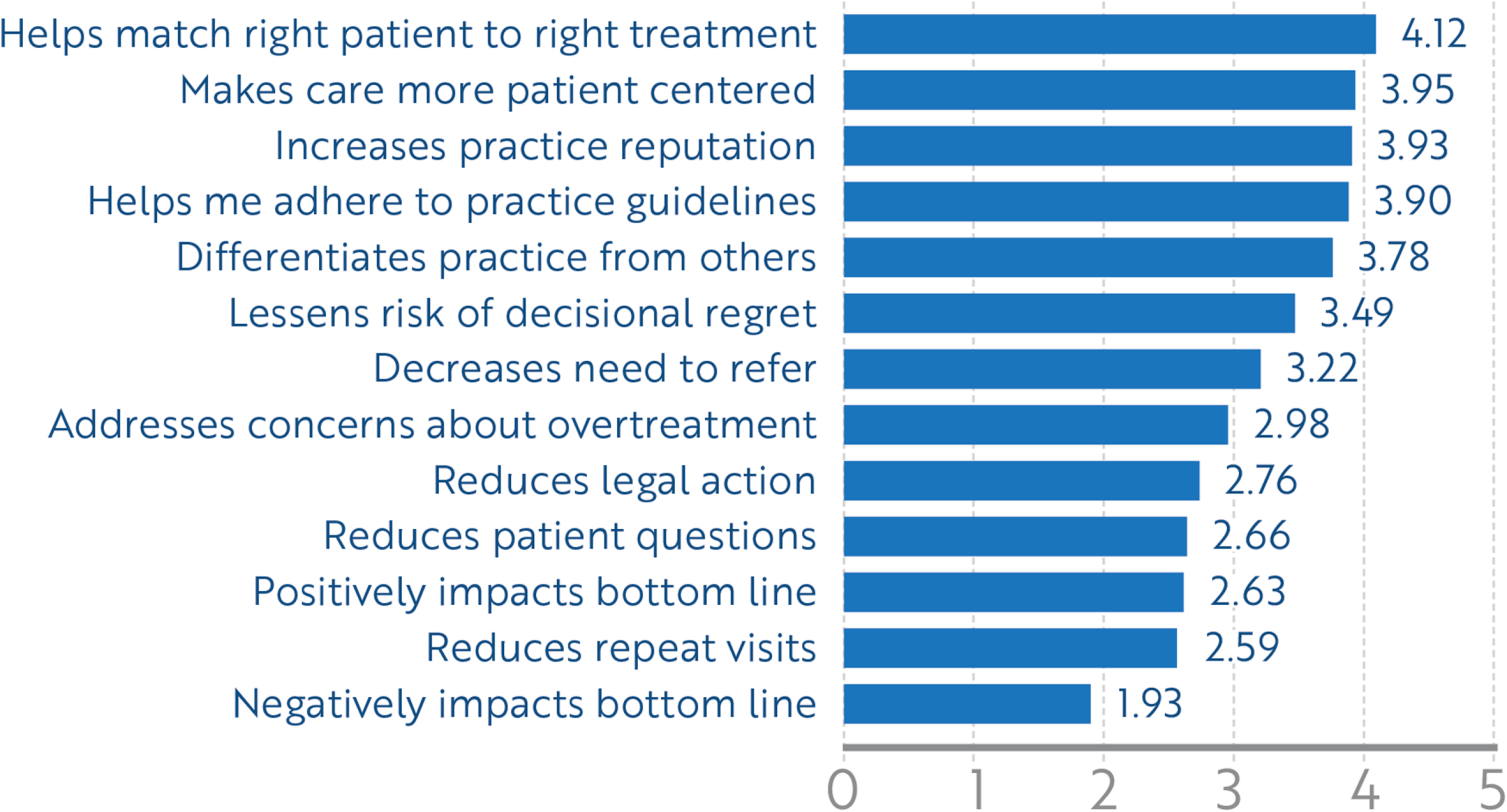
Average rating of innovation adoption attributes across the sample, *n* = 54. Higher scores indicate greater agreement

Top-rated innovation attributes (Fig. 3) were: (1) helping the urologist to match the right patient to right treatment; (2) increasing a practice’s reputation as offering cutting-edge treatment options; (3) helping to make care more patient-centered; (4) helping the urologist to adhere to practice guidelines; (5) differentiating the urologists’ practice from other practices; (6) lessening the patients’ risk of decisional regret; and (7) decreasing the need to refer. No statistically significant differences were found between those who offered trials and those who did not among any of the innovation attributes. After urologists ranked their top three innovation attributes, 43% of urologists included practice reputation in their top three reasons for offering clinical trials; 30% listed practice differentiation among their top three reasons.

## Discussion

We developed an implementation intervention that is viewed as highly acceptable and appropriate to professionally engaged urologists, a majority of whom indicated an intention to adopt the program. The implementation intervention potentially has broad reach, as our participants represented both urban and rural, and community and academic practices across a sizable geographic portion of North America. Confirming our implementation intervention design, which underscored practice differentiation as a key motivator, participants highly valued innovations which would set them apart from other providers. Further, our design of the program to position the offer of clinical trials as an expansion of treatment options was validated by urologists’ high ratings of matching the right patient to the right treatment as an appealing innovation attribute. Because implementation intervention users have defined this idea of “precision medicine” as an expectation for an innovation that they are willing to adopt, any future implementation intervention effectiveness assessments should include the approach’s ability to deliver on this expectation.

The program we designed is unique in multiple ways among strategies to increase the offer of clinical trials. First, it focuses on the structural disjuncture in the care delivery environment that separates urological cancer patients from several guideline-based cancer services, including clinical trials. In this way, it is designed to increase the reach of clinical trials, a critical component of intervention effectiveness [55]. Increasing reach may be particularly important in the current era of cancer clinical trials, where precision medicine trials requiring higher levels of population screening to identify eligible participants are gaining dominance [56, 57]. Second, it leverages the existing professional identities of community-based urologists. Rather than requiring practices to provide the infrastructure to open trials in their own practice, such as study personnel and IRB regulatory requirements, it allows them to capitalize on natural referral behaviors and expands their networks to include trial experts. Other programs, such as the Society of Urological Oncology-Clinical Trials Consortium, cater to larger practices that can invest in research infrastructure [22]. While successful, not all practices can provide this level of skill and capacity to meet guideline recommendations. The National Cancer Institute’s Community Oncology Research Program, although effective in expanding trial options to community providers [1, 58], focuses on oncology practices [16] and the degree to which they effectively extend to non-oncology cancer providers is not well understood [59]. Other initiatives mainly offer educational and navigational support for patients [23, 24, 60–62] and rely on patient activation in a patient-physician dynamic which weighs heavily toward physician influence. Without recommendation of the physician, few patients will participate in trials [6, 63]. Finally, our intervention is unique because, to our knowledge, it is the first to apply principles of implementation science to the decades-old, intransigent problem of low accrual to clinical trials. Continued research to assess its effectiveness can further the developing field. Although the implementation intervention was highly rated, we observed some differentiation in its acceptability ratings between urologists experienced in offering clinical trials and research-naïve urologists. While both rated the implementation intervention as highly acceptable, those with experience rated it significantly higher. Although the instrument used to measure acceptability has been validated [64], this is the first study, to our knowledge, to provide scores for a population of potential adopters. Thus, scores have not been normalized among other interventions or adopters, providing little context in which to interpret either the ratings observed in this study or their differentiation among users. Future studies should compare ratings against those found in this study. Nonetheless, we observed no differentiation in ratings of appropriateness or individual attributes of innovation appeal, suggesting that, perhaps, in this sample of urologists, trial-naïve participants thought that they *should* offer clinical trials, but may still see components of the implementation intervention as not completely aligning with their practice ecology.

Moreover, there may be some motivational goals that we have not identified. Attributes included in the measure were identified from qualitative interviews with urologists around treatment decision making in prostate cancer and in the context of offering clinical trials [47]. The items remain to be validated in a larger sample of providers. However, participants provided no additional innovation attributes that promote adoption when afforded the opportunity.

Our implementation intervention is designed on some key assumptions. One is that community urologists are not currently engaging their patients about clinical trials. Although Ellis et al. reported recent qualitative data that supports this [19], the last nationally representative survey of urologists was conducted in 2005. By approaching urologists through state professional societies, we reached more providers who self-reported that they already offered clinical trials than those who self-identified as trial-naive. Thus, contemporary assessment of urologists’ involvement in clinical trials is needed. Changes in both the clinical trial landscape (i.e., growth of precision medicine trials, increased presence of National Cancer Institute Community Oncology Research Program (NCORP)) and urological cancer care delivery (e.g., continued consolidation of practices, decline in solo practice, shortages of common bladder cancer therapies currently in clinical trial) may have impacted urologists’ engagement in trials. One opportunity is to query the NCI’s Community Oncology Research Program to identify how many cancer components already include urologists.

Finally, we report potential adopters’ assessment of the acceptability, appropriateness and appeal of the implementation intervention, we did not test feasibility among urology practices, effectiveness of the approach in increasing referrals or accrual, or, importantly, the acceptability of the approach to cancer programs that are essential partners in the implementation approach. Academic physicians and cancer center administrators and staff will be critical partners supporting community physicians’ offer of trials. Thus, it is essential to assess these partners’ engagement, and willingness to participate, and to identify what successful strategies have already been tried [65]. Further, future research should address the degree to which cancer programs offering clinical trials would welcome additional eligibility screening responsibilities and measure the additional workload required.

### Limitations

Our study is not without limitations. We developed and tested the implementation intervention across the Midwest and select Southern states and found it to be appealing. However, results may not be generalizable. Whether the appeal of the implementation intervention extends to other practices awaits future opportunities to offer the program in other regions of the country. Efforts to extend the implementation intervention to other regions of the country are ongoing and should be rigorously tested with the appropriate study design. A cluster-randomized trial can assess whether an intervention informed by principles of implementation science can increase the rate of cancer patients’ participation. Secondly, our rigorous, theoretically based implementation development process should promote rapid adoption and implementation of the program. However, both whether the program will be adopted and the predictive value of acceptability and appropriateness ratings on subsequent adoption should be measured.

Finally, the application of the Behavior Change Wheel is subjective and resulted in a large number of intervention components. Although the framework is theoretically and empirically derived, mapping of behavior-change techniques to specific determinants of behavior is not yet an exact science. A great deal of heterogeneity in mapping the most appropriate technique to a specific determinant has been observed [66]. Thus, others may have categorized determinants, functions, or behavior-change techniques differently. Members of our team have experience with determinant coding and some have training in behavior-change technique coding, which should promote concordance. Nonetheless, the inability to empirically map the behavior-change techniques to determinants underscores the need for effectiveness testing. Additional implementation research can identify the core components of the implementation intervention and inform future efforts to optimize those implementation strategies.

## Conclusions

Low-enrolling studies slow the uptake of innovations in cancer care [2, 58], and are costly to study sponsors and the institutions that conduct them [67]. We have developed a promising implementation intervention to address this problem in an understudied segment of cancer providers. The implementation intervention is appealing to its target audience. Implementation intervention effectiveness and the effect of its acceptability, appropriateness and appeal on the adoption of the offer of clinical trials should be further tested.

## Additional file

Additional file 1: LEARN|INFORM|RECRUIT Appeal Survey. (DOCX 17 kb)

## Data Availability

The dataset created and analyzed during the current study is available from the corresponding author on reasonable request.

## Abbreviations

AIM: Acceptability of Intervention Measure
AUA: American Urological Association
IAM: Intervention Appropriateness Measure
NCI: National Cancer Institute
SCS: South Central Section (of the American Urological Association)
TDF: Theoretical Domains Framework
