# Supplementary material for: Development, acceptability, appropriateness and appeal of a cancer clinical trials implementation intervention for rural- and minority-serving urology practices
Source: Trials. 2019 Oct 7;20:578. doi: 10.1186/s13063-019-3658-z (PMC6781342; doi:10.1186/s13063-019-3658-z)
Supplement: Supplementary file 1 — LEARN|INFORM|RECRUIT Appeal Survey. (DOCX 17 kb) [file 13063_2019_3658_MOESM1_ESM.docx]

**LEARN INFORM RECRUIT Advisory Panel**

**Curriculum Feedback**

**v. 2/2/18**

Thank you for watching our program on offering clinical trials in your urology practice. We are trying to understand how community urologists might find this program useful. Please complete the following questions by rating the acceptability of the LEARN INFORM RECRUIT educational activity for practicing urologists.

1. **Please select your level of agreement by marking the appropriate circle. ***

|  | Completely disagree | Disagree | Neither agree nor disagree | Agree | Completely agree |
| --- | --- | --- | --- | --- | --- |
| The LEARN\|INFORM\|RECRUIT educational activity meets my approval. | ➀ | ➁ | ➂ | ➃ | ➄ |
| The LEARN\|INFORM\|RECRUIT educational activity is appealing to me. | ➀ | ➁ | ➂ | ➃ | ➄ |
| I like the LEARN\|INFORM\|RECRUIT educational activity. | ➀ | ➁ | ➂ | ➃ | ➄ |
| I welcome the LEARN\|INFORM\|RECRUIT educational activity. | ➀ | ➁ | ➂ | ➃ | ➄ |

1. **For any item that you marked disagree or completely disagree, please describe why you did not rank the LEARN INFORM RECRUIT activity higher:**
2. **Please complete the following questions by rating the appropriateness of the LEARN INFORM RECRUIT educational activity for presentation at a professional society meeting.***

|  | Completely disagree | Disagree | Neither agree nor disagree | Agree | Completely agree |
| --- | --- | --- | --- | --- | --- |
| The LEARN\|INFORM\|RECRUIT educational activity seems fitting. | ➀ | ➁ | ➂ | ➃ | ➄ |
| The LEARN\|INFORM\|RECRUIT educational activity seems suitable. | ➀ | ➁ | ➂ | ➃ | ➄ |
| The LEARN\|INFORM\|RECRUIT educational activity seems applicable. | ➀ | ➁ | ➂ | ➃ | ➄ |
| The LEARN\|INFORM\|RECRUIT educational activity seems like a good match. | ➀ | ➁ | ➂ | ➃ | ➄ |

1. **For any item that you marked disagree or completely disagree, please describe why you did not rank the LEARN INFORM RECRUIT activity higher:**
2. **Do you currently offer clinical trials to your patients?** ___Yes ___No
3. **Urologists consider a variety of things about new treatment options before adopting them. Please indicate how offering clinical trials relates to your practice priorities. Circle the response corresponding to the level of importance you place on each attribute.**

|  | Not at all important | Slightly important | Moderately important | Very important | Extremely important |
| --- | --- | --- | --- | --- | --- |
| 1. Offering clinical trials help me match the right patient to the right treatment | ➀ | ➁ | ➂ | ➃ | ➄ |
| 1. Offering clinical trials reduces vulnerability to legal action | ➀ | ➁ | ➂ | ➃ | ➄ |
| 1. Offering clinical trials lessens the risk of patients' decisional regret | ➀ | ➁ | ➂ | ➃ | ➄ |
| 1. Offering clinical trials helps me adhere to practice guidelines | ➀ | ➁ | ➂ | ➃ | ➄ |
| 1. Offering clinical trials reduces patient questions | ➀ | ➁ | ➂ | ➃ | ➄ |
| 1. Offering clinical trials reduces repeat visits to discuss treatment options | ➀ | ➁ | ➂ | ➃ | ➄ |
| 1. Offering clinical trials increases my practice's reputation as offering cutting edge treatment options | ➀ | ➁ | ➂ | ➃ | ➄ |
| 1. Offering clinical trials decreases my need to refer patients to other healthcare providers | ➀ | ➁ | ➂ | ➃ | ➄ |
| 1. Offering clinical trials negatively impacts my practice’s bottom line | ➀ | ➁ | ➂ | ➃ | ➄ |
| 1. Offering clinical trials positively impacts my practice’s bottom line | ➀ | ➁ | ➂ | ➃ | ➄ |
| 1. Offering clinical trials differentiates my urology practice from other specialty practices in the area | ➀ | ➁ | ➂ | ➃ | ➄ |
| 1. Offering clinical trials addresses public concerns about overtreatment |  |  |  |  |  |
| 1. Offering clinical trials making care more patient centered |  |  |  |  |  |
| 1. Other (_______________________________________________) |  |  |  |  |  |

1. **Among all 14 attributes, rank three that are most important to you (write attribute number in spaces below).**
2. ____________________________ 2. _____________________________ 3. ____________________________

* Acceptability of Intervention Measure used with permission; Weiner et al, 2017 *Implementation Science 12:108*

**Intervention Appropriateness Measure used with permission; Weiner et al, 2017 *Implementation Science 12:108*
